# Internalized PCSK9 dissociates from recycling LDL receptors in PCSK9-resistant SV-589 fibroblasts

**DOI:** 10.1194/jlr.M044156

**Published:** 2014-02

**Authors:** My-Anh Nguyen, Tanja Kosenko, Thomas A. Lagace

**Affiliations:** Department of Pathology and Laboratory Medicine, University of Ottawa Heart Institute, Ottawa, Ontario, Canada K1Y 4W7

**Keywords:** endosome, degradation, ligand dissociation

## Abstract

Secreted PCSK9 binds to cell surface LDL receptor (LDLR) and directs the receptor for lysosomal degradation. PCSK9 is potent at inducing LDLR degradation in cultured liver-derived cells, but it is considerably less active in immortalized fibroblasts. We examined PCSK9 trafficking in SV-589 human skin fibroblasts incubated with purified recombinant wild-type PCSK9 or gain-of-function mutant PCSK9-D374Y with increased LDLR binding affinity. Despite LDLR-dependent PCSK9 uptake, cell surface LDLR levels in SV-589 fibroblasts were only modestly reduced by wild-type PCSK9, even at high nonphysiological concentrations (20 µg/ml). Internalized ^125^I-labeled wild-type PCSK9 underwent lysosomal degradation at high levels, indicating its dissociation from recycling LDLRs. PCSK9-D374Y (2 µg/ml) reduced cell surface LDLRs by approximately 50%, but this effect was still blunted compared with HepG2 hepatoma cells. Radioiodinated PCSK9-D374Y was degraded less efficiently in SV-589 fibroblasts, and Alexa488-labeled PCSK9-D374Y trafficked to both lysosomes and endocytic recycling compartments. Endocytic recycling assays showed that more than 50% of internalized PCSK9-D374Y recycled to the cell surface compared with less than 10% for wild-type PCSK9. These data support that wild-type PCSK9 readily dissociates from the LDLR within early endosomes of SV-589 fibroblasts, contributing to PCSK9-resistance. Although a large proportion of gain-of-function PCSK9-D374Y remains bound to LDLR in these cells, degradative activity is still diminished.

The low density lipoprotein receptor (LDLR) binds with high affinity to the apolipoprotein (apo)B100 and apoE components of circulating LDL and VLDL, respectively, via a ligand-binding domain consisting of seven cysteine-rich repeats (R1–R7) (1). Following LDLR-mediated endocytosis, bound lipoproteins are released within the acidic environment of early endosomes and the LDLR recycles to the cell surface. Lipoprotein release and LDLR recycling are facilitated by a pH-dependent open (ligand-active) to closed (ligand-inactive) conformational switch involving an intramolecular interaction between the R4/R5 repeats of the ligand-binding domain and a β-propeller region in the epidermal growth factor (EGF) precursor homology domain of LDLR (2, 3). LDLR-mediated uptake of LDL in the liver is the primary means of plasma LDL clearance and thus influences steady-state plasma LDL-cholesterol (LDL-C) levels and associated risk of coronary artery disease (4).

LDLR also binds proprotein convertase subtilisin/kexin type-9 (PCSK9), a secreted member of the mammalian proprotein convertase family of serine endoproteases (5–7). Bound PCSK9 disrupts the normal endocytic recycling itinerary of the LDLR and directs the receptor for degradation in endolysosomal compartments (8–11). PCSK9 binds in a Ca^2+^-dependent manner to the first of three Ca^2+^-binding EGF-like repeats (EGF-A) in the EGF precursor homology domain of LDLR and demonstrates increased binding affinity at acidic pH (9, 12, 13). Gain-of-function mutations in *PCSK9* are associated with autosomal dominant hypercholesterolemia (14, 15), with the missense D374Y variant causing a particularly severe form of the disease (16). The D374Y substitution in the catalytic domain of PCSK9 improves a key bonding interaction with H306 in the LDLR EGF-A domain resulting in 10- to 25-fold increased binding affinity at both neutral and acidic pH (17–19). Loss-of-function mutations in *PCSK9* are relatively common among certain ethnic groups and are associated with lowered plasma LDL-C and significant protection from coronary artery disease (20, 21). Antagonism of circulating PCSK9 using injectable anti-PCSK9 monoclonal antibodies that disrupt binding to LDLR resulted in substantial LDL-C lowering in Phase II clinical trials (22–24), supporting that secreted PCSK9 is a main regulator of circulating LDL-C levels in humans.

PCSK9 is primarily expressed and secreted from liver, with lower levels of expression in kidney, intestine and brain (25). PCSK9 is initially synthesized as a soluble 74 kDa precursor that undergoes autocatalytic cleavage in the ER lumen, releasing an approximately 14 kDa prodomain segment, which noncovalently associates with the approximately 60 kDa catalytic/C-terminal domains of PCSK9 and acts as a folding chaperone and inhibitor of inherent protease activity (8). The prodomain remains tightly bound within the catalytic pocket and as a consequence mature secreted PCSK9 is catalytically inert (12, 13). Catalytic activity is not required for PCSK9 to direct LDLR degradation in hepatic cells in culture or in mouse liver (26, 27). While the precise mechanism remains undefined, it is theorized that PCSK9 binding to the EGF-A domain inhibits the acid-dependent open-to-closed conformational switch of the LDLR in early endosomes, making the receptor more prone to lysosomal sorting mechanisms or proteolytic attack (28–30). In addition to the established binding interface between the PCSK9 catalytic domain and LDLR EGF-A domain, deletion mutagenesis and cellular LDLR degradation studies have revealed requirements for the C-terminal domain of PCSK9 and at least three LDLR ligand binding repeats, suggesting the involvement of these domain regions in structural aspects of LDLR degradation or additional protein-protein interactions (28, 31–34).

Although highly active in liver-derived cells in culture, exogenous PCSK9 is much less potent at directing LDLR degradation in immortalized fibroblasts, despite internalization along with LDLRs into endosomal compartments (5, 35). To explore mechanisms of intracellular PCSK9 resistance, we examined LDLR-dependent uptake and trafficking of PCSK9 in SV-589 cells, a line of SV40-transformed human skin fibroblasts shown to be highly resistant to PCSK9-mediated LDLR degradation (35).

## MATERIALS AND METHODS

### Materials

We obtained fetal bovine serum (FBS), newborn calf serum, human transferrin and Lipofectamine 2000 from Life Technologies. E64 (N-[N-(L-3-trans-carboxyoxirane-2-carbonyl)-L-leucyl]agmatine) and EDTA-free Complete^TM^ Protease Inhibitor Tablets were obtained from Roche. PureProteome^TM^ Streptavidin Magnetic Beads from Millipore. IRDye800CW Streptavidin was from LI-COR Biosciences. Na ^125^I was from PerkinElmer. Cholesterol and 25-hydroxycholesterol were purchased from Steroloids, and all other chemicals and reagents from Sigma unless otherwise specified. LDLR cDNA expression vector was pLDLR17 (36). Sodium mevalonate was prepared from mevalonic acid as described (37). Newborn calf lipoprotein-deficient serum (NCLPDS) (*d* > 1.215 g/ml) was prepared by ultracentrifugation (38).

### Antibodies

rabbit anti-serum 3143 against the C-terminal 14 amino acids of the LDLR was the kind gift of J. Herz (University of Texas Southwestern Medical Center, Dallas, TX); C7 antibody was purified from conditioned medium of mouse hybridoma cells (ATCC, CRL-1691) by protein A affinity chromatography using Profinia^TM^ affinity chromatography purification system (Bio-Rad); mouse anti-human transferrin receptor antibody was purchased from Life Technologies; monoclonal anti-FLAG M2 antibody were from Sigma-Aldrich. Secondary IRDye-labeled goat anti-mouse and anti-rabbit IgG antibodies were from LI-COR Biosciences.

### Protein purification and labeling

FLAG epitope-tagged recombinant human wild-type PCSK9 and PCSK9-D374Y were purified as previously described (39). PCSK9 was labeled with the AlexaFluor488 Protein Labeling Kit (Life Technologies) as per manufacturer's protocol followed by gel filtration chromatography on a Superdex 200 10/300 GL column (GE Healthcare) to remove unbound dye. PCSK9 was labeled with EZ-Link^TM^ Sulfo-NHS-SS-Biotin (Thermo Scientific Pierce) according to the manufacturer's protocol. Free biotin was quenched in Tris-glycine buffer (25 mM Tris-HCl, pH 7.4; 192 mM Glycine) and removed from biotinylated PCSK9 by gel filtration.

### Radiolabeling of proteins

Purified wild-type PCSK9, PCSK9-D374Y or C7 monoclonal antibody [400 µg in 200 µl Hepes-buffered saline containing 2 mM CaCl_2_, pH 7.4 (HBS-C)] were incubated with carrier-free Na ^125^I (2 mCi) in Precoated Iodination Tubes (Thermo Scientific Pierce) for 10 min. The reaction was stopped by removal to a glass test tube and brought to a 500 µl volume in scavenging buffer (HBS-C containing 1 mM NaI carrier and 2 mg/ml tyrosine) and incubated 5 min. Free ^125^I and scavenged ^125^I were removed from iodinated proteins by gel filtration on a PD10 column (GE Healthcare) equilibrated in HBS-C. Integrity of iodinated proteins was confirmed by SDS-PAGE and autoradiography of dried gels. Radiolabeled proteins were stored at 4°C and used within two weeks.

### Cultured cell experiments

SV-589 human skin fibroblast cells (kindly provided by J. Goldstein, University of Texas Southwestern Medical Center, Dallas, TX) and HepG2 human hepatoma cells (ATCC, HB-8065) were maintained in monolayer culture at 37°C and 5% CO_2_ (SV-589) or 8.8% CO_2_ (HepG2). The base medium was DMEM (Gibco-Life Technologies) containing 4.5 g/L glucose for SV-589 cells or DMEM containing 1 g/L glucose for HepG2 cells. Medium A contained DMEM supplemented 100 U/ml penicillin and 100 µg/ml streptomycin sulfate; Medium B contained Medium A supplemented with 10% FBS (v/v); sterol-depleting Medium C contained Medium A supplemented with 5% (v/v) NCLPDS, 10 µM pravastatin, and 50 µM sodium mevalonate; sterol-supplemented Medium D contained Medium A with 5% (v/v) NCLPDS, 10 µg/ml cholesterol, and 1 µg/ml 25-hydroxycholesterol. Cells were plated and grown in Medium B to approximately 60% confluency prior to start of experiments.

### Cell surface biotinylation and immunoblotting

SV-589 and HepG2 cells were cultured overnight in sterol-depleting or sterol-supplemented media conditions prior to treatment with PCSK9 (see figure legends for details). Following treatments, cells were scraped and collected in PBS and whole-cell extracts were prepared in Tris lysis buffer [50 mM Tris-Cl, pH 7.4; 150 mM NaCl; 1% Nonidet P-40 (EMD Biosciences); 0.5% sodium deoxycholate; 5 mM EDTA; 5 mM EGTA; Complete^TM^ protease inhibitor cocktail; 1 mM phenylmethylsulfonyl fluoride (PMSF)]. For some experiments, cell surface proteins were biotinylated prior to harvesting using EZ-Link Sulfo-NHS-SS-Biotin (Thermo Scientific Pierce) as described (5). Three quarters of each cell lysate were brought up to 0.5 ml in Tris lysis buffer and rotated overnight at 4°C with streptavidin magnetic beads. The beads were washed three times with Tris-lysis buffer and protein eluted in 1× SDS loading buffer (50 mM Tris-HCl, pH 6.8; 1% SDS; 5% glycerol; 10 mM EDTA; 0.0032% bromophenol blue). Precipitated cell surface protein and whole-cell extract proteins were subjected to 8% SDS-PAGE, transferred to nitrocellulose membrane (Bio-Rad) and incubated with primary antibodies. Infrared dye (IRDye-800)-labeled secondary antibodies were used for detection on LI-COR Odyssey infrared system (LI-COR Biosciences). Band intensity was analyzed using Odyssey 2.0 software.

### Flow cytometry

SV-589 and HepG2 cells were cultured overnight in sterol-depleting Medium C, respectively, containing 150 µM E64, a noncell-permeable version of a cysteine protease inhibitor shown to inhibit lysosomal degradation of internalized PCSK9 in cultured cells (35). Following 1 h incubation with Alexa488-labeled PCSK9-D374Y (1 μg/ml), cells were washed with stripping buffer (100 mM Na 2-mercapto-ethanesulfonate; 50 mM Tris, pH 8.6; 100 mM NaCl; 1 mM EDTA; 0.2% BSA) to remove cell surface-associated fluorescence. Harvested cells were filtered through a 70 μM cell strainer (BD Biosciences) and subjected to flow cytometry using a BD FACSAria flow cytometer and cell sorter (BD Biosicences). The lower threshold of positive staining was determined using cells not incubated with fluorescent protein.

### ^125^I-PCSK9 degradation assay

SV-589 and HepG2 cells were cultured overnight in sterol-depleting Medium C, respectively, prior to pulse-labeling with ^125^I-labeled wild-type PCSK9 (5 µg/ml), PCSK9-D374Y (0.5 µg/ml), or C7 antibody (0.5 µg/ml) for 1 h in Medium A containing 5% NCLPDS. In control experiments, cells were pretreated for 30 min and then throughout with 50 µM chloroquine to inhibit lysosomal degradation. Following labeling, cells were washed and incubated in label-free Medium A with 5% NCLPDS for 6 h at 37°C. Medium was collected and total protein was precipitated on ice with 10% (v/v) trichloroacetic acid (TCA), and pelleted by centrifugation. TCA-soluble supernatant (1 ml) was removed to glass tubes, and mixed with 10 μl of 40% KI followed by 40 μl of 30% hydrogen peroxide. Reactions were allowed to stand for 10 min then extracted with 2 ml of chloroform to remove free iodide. A 700 μl of the upper aqueous phase containing ^125^I-monoiodotyrosine was removed for γ counting using a Cobra^TM^ II Auto-Gamma counter. Measurements were normalized to cell protein levels determined by BCA assay.

### Live cell imaging and colocalization

SV-589 cells were cultured on Lab-Tek (Nunc) 8-well chambered coverglass and incubated overnight in sterol-depleting Medium C containing 150 μM E64. For colocalization of Alexa488-labeled PCSK9 with LysoTracker Red DND-99 (Life Technologies) cells were labeled with Alexa488-labeled wild-type PCSK9 (30 µg/ml) or D347Y PCSK9 (5 µg/ml) for 1 h and chased for up to 6 h in label-free Medium C containing E64. LysoTracker Red DND-99 was incubated at a concentration 200 nM for 2 h prior to the end of each chase period. For colocalization studies of Alexa488-labeled PCSK9 and Alexa647-labeled transferrin, cells were preincubated for a minimum 1 h in serum-free Medium A to deplete endogenous transferrin. Cells were then incubated with Alexa488-labeled wild-type PCSK9 (30 µg/ml) or PCSK9-D347Y (5 µg/ml), and Alexa647-labeled transferrin (100 µg/ml) for 1 h in Medium A. The labeled proteins were chased for 2 h in label-free Medium A containing E64, then visualized directly. Images were taken on an Olympus FV1000 scanning confocal microscope. Colocalization was quantified using Image J (http://rsb.info.nih.gov/ij/).

### Endocytic recycling assay

SV589 cells seeded in 24-well plates were cultured overnight in Medium C containing 150 μM E64. PCSK9 labeled with thiol-cleavable biotin was incubated with IRDye800CW-labeled streptavidin for 1 h at 37°C, and the complexes were then added to the cells. After 1 h, the cells were incubated with 20 mM Tris(2-carboxyethyl)phosphine (TCEP) in buffer B (PBS, 0.1 mM CaCl_2_, 2 mM MgCl_2_, 0.5% BSA (w/v)) for 20 min at 4°C, then washed twice for 10 min with 5 mg/ml iodoacetamide in buffer B. The cells were washed again with buffer B and PBS-CM (PBS, 0.1 mM CaCl_2_, 2 mM MgCl_2_), then incubated in Medium A, supplemented with TCEP and E64, for 6 h at 37°C. The plate was directly scanned on the LI-COR Odyssey infrared system at time intervals. Signal intensity was quantified using Odyssey 2.0 software, background fluorescence of untreated wells was subtracted, and signal normalized to DNA levels stained by DRAQ5 emitting at a separate wavelength (Cedarlane Laboratories, Canada) (1:10,000).

### Ligand blotting

HEK293 cells cultured in Medium B were transiently transfected with pLDLR17 vector using Lipofectamine 2000 (Life Technologies). After 48 h cells were scraped and pelleted in cold PBS then resuspended on ice in sorbitol microsome buffer (50 mm HEPES-KOH, pH 7.2, 250 mm sorbitol, 10 mm KCl, 1.5 mm MgCl_2_, Complete^TM^ protease inhibitor mixture, 1 mm PMSF) and disrupted with 10 passages through a 23-gauge needle. Membranes were pelleted by centrifugation at 100,000 × *g* for 30 min then resuspended in pH 6/Triton X-100 buffer (40 mm Tris-maleate, pH 6.0, 100 mm NaCl, 2 mm CaCl_2_, 1 mm MgCl_2_, 1% Triton X-100, Complete^TM^ protease inhibitor mixture, 1 mm PMSF). Membrane proteins (35 μg/lane) were resolved on 8% SDS-PAGE in nonreducing loading buffer (50 mM Tris-maleate, pH 6.0, 2 mM CaCl_2_, 0.5% SDS, 10% glycerol, 0.05% bromophenol blue) and transferred to nitrocellulose membranes. Membranes was cut in strips and blocked for 30 min in blocking buffer (50 mm Tris-Cl, pH 7.0, 90 mm NaCl, 2 mm CaCl_2_, 5% (w/v) skim milk), then incubated in blocking buffer with ^125^I labeled wild-type PCSK9-FLAG (10 µg/ml) or PCSK9-D374Y (2 µg/ml) in the absence or presence of 50 µg/ml of unlabeled PCSK9-D374Y. Membranes were washed three times in blocking buffer and visualized on a Storm 860 Molecular Imager (GE Healthcare).

### Statistical analysis

All presented values are mean and standard deviation. Statistical analysis was determined by Student *t*-test with GraphPad Prism 5 software.

## RESULTS

### Limited degradation of LDLRs in SV-589 fibroblasts in response to high PCSK9 concentrations

**Fig. 1.**
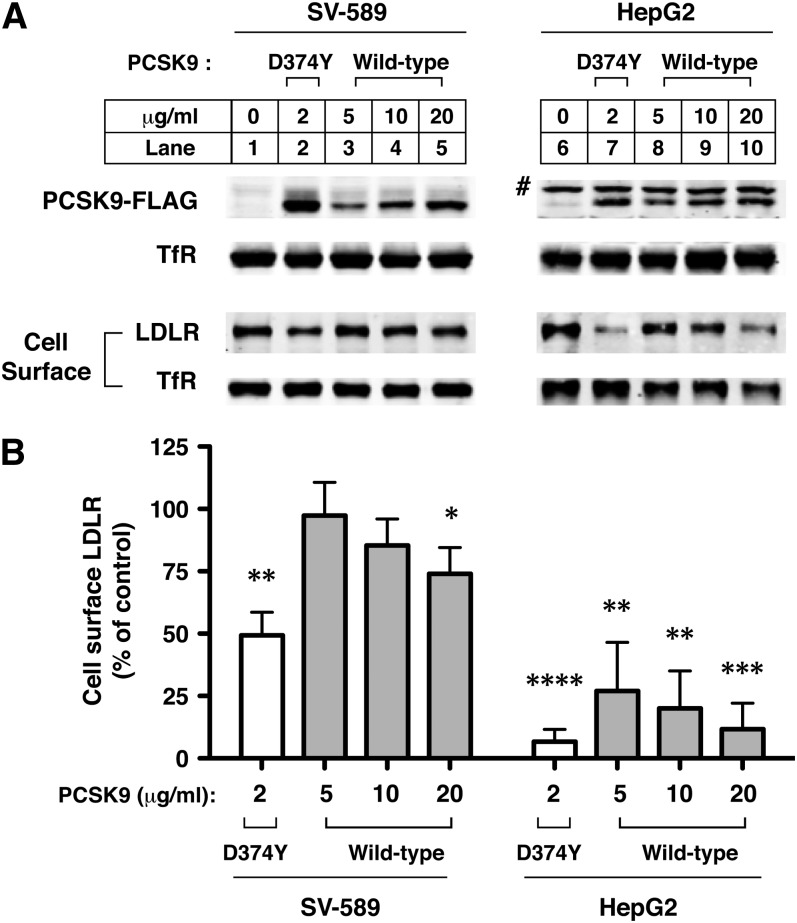
Decreased ability of PCSK9 to mediate LDLR degradation in SV-589 fibroblasts. A: SV-589 and HepG2 cells were cultured for more than 16 h in sterol-depleting Medium C prior to incubation with indicated concentrations of purified FLAG-tagged PCSK9 or PCSK9-D374Y for 6 h at 37°C. Cell surface proteins were biotinylated and whole-cell and cell surface extracts were subjected to SDS-PAGE and immunoblot detection of LDLR and the FLAG-epitope on PCSK9. TfR was detected as a control for loading and nonspecific protein degradation. Infrared dye (IRDye800)-labeled secondary antibodies were used for imaging on a LI-COR Odyssey infrared system. **#** indicates nonspecific protein recognized by anti-FLAG M2 antibody in HepG2 cells. B: Quantification of cell surface LDLR normalized to TfR levels. Average and standard deviation of four separate experiments is shown. *P < 0.05, **P < 0.01, ***P < 0.005, ****P < 0.001 compared with untreated control (Student *t*-test).

PCSK9 concentrations in human plasma range from 0.05 to 3 μg/ml among healthy individuals (40). A recent study showed that SV-589 cells, a line of SV40-transformed human skin fibroblasts, showed no decrease in LDLR levels in response to a 4 h treatment with 10 μg/ml concentration of purified PCSK9, whereas this same treatment decreased LDLR levels in HuH7 hepatoma cells and primary human fibroblasts (35). To further assess the extent of PCSK9 resistance in SV-589 fibroblasts, we incubated cells with purified recombinant wild-type PCSK9 at high nonphysiological concentrations (5–20 µg/ml) or with a lower concentration (2 µg/ml) of a gain-of-function PCSK9 variant (PCSK9-D374Y) that has increased LDLR affinity. As a comparison, HepG2 hepatoma cells were treated under identical conditions. Cells were first cultured for more than 16 h in lipoprotein-deficient medium containing a statin (pravastatin) to induce LDLR mRNA and protein expression. This culture condition also induced endogenous PCSK9 expression and secretion in both cell-lines (data not shown). To eliminate possible interference by secreted endogenous PCSK9, culture medium was replaced immediately prior to 6 h incubation with FLAG-tagged wild-type PCSK9 or PCSK9-D374Y. Cells were harvested and immunoblot analysis was performed to determine PCSK9 and LDLR levels present in whole cell extracts and among cell surface proteins isolated following cell surface biotinylation. As expected, treatment with PCSK9 greatly reduced cell surface LDLR expression in HepG2 hepatoma cells, whereas SV-589 cells remained highly resistant to LDLR degradation, despite similar levels of cell-association of FLAG-tagged PCSK9 (**Fig. 1A**). While there was a trend toward dose-dependent reduction of SV-589 cell surface LDLR in response to high concentrations of wild-type PCSK9, this effect only reached statistical significance at the highest PCSK9 concentration tested (20 µg/ml), which reduced cell surface LDLR levels by approximately 25% (Fig. 1B). We obtained similar results following longer 18 h incubations with PCSK9 (data not shown), ruling out the possibility that resistance to PCSK9-mediated LDLR degradation observed in SV-589 fibroblasts was due to slower rates of cell surface LDLR endocytosis compared with HepG2 cells. Gain-of-function PCSK9-D374Y at a concentration of 2 µg/ml significantly reduced LDLR levels on the cell surface (>50%) in SV-589 fibroblasts (Fig. 1B), although this response was still much weaker than that seen in HepG2 cells. Thus, immortalized SV589 fibroblasts do not display absolute resistance to PCSK9-mediated LDLR degradation and are susceptible to moderately elevated concentrations of the gain-of-function mutant PCSK9-D374Y.

### Sterol-dependent uptake of PCSK9 in SV-589 fibroblasts

**Fig. 2.**
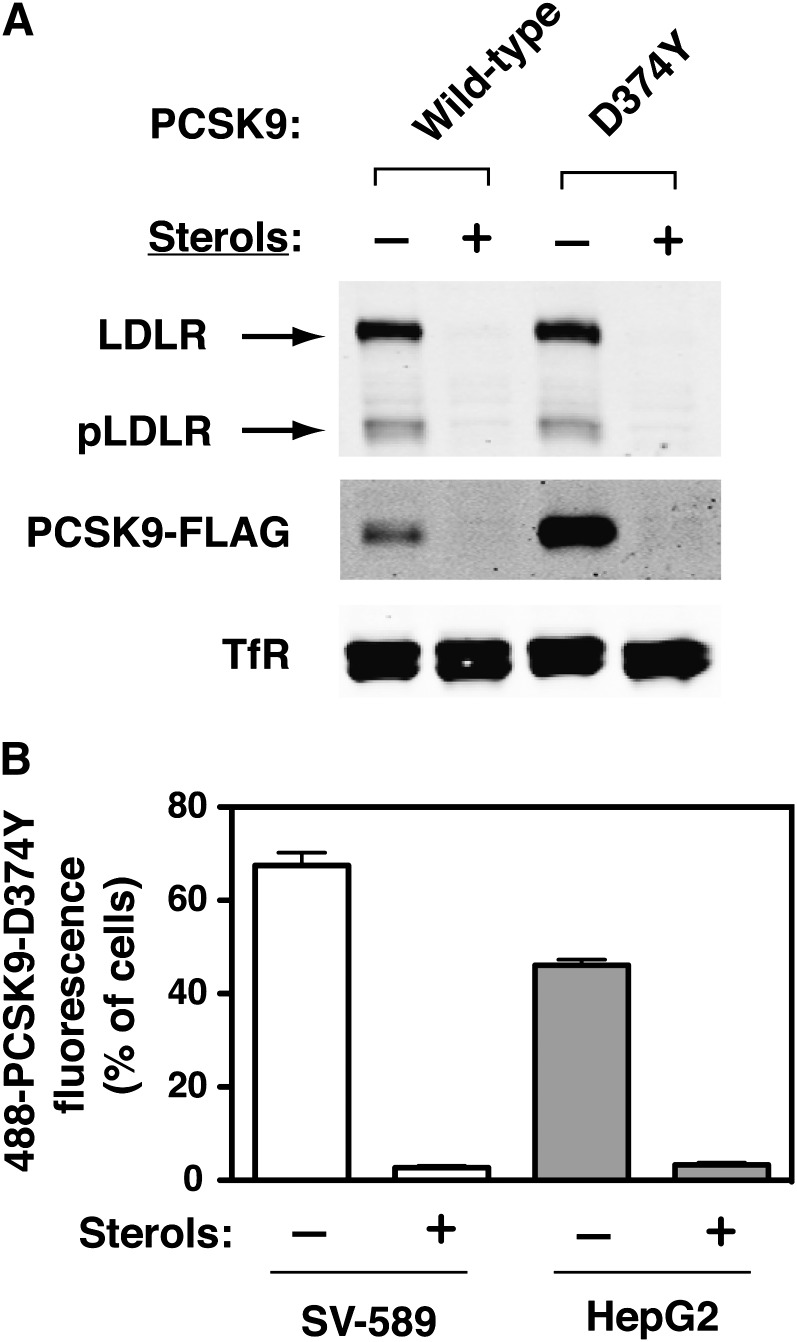
Sterol-dependent cell association and uptake of PCSK9. A: SV-589 fibroblasts were cultured for more than 16 h in the absence (−) or presence (+) of sterols as described in Materials and Methods prior to 2 h treatment with purified FLAG-tagged wild-type PCSK9 (5 µg/ml) or PCSK9-D374Y (0.5 µg/ml). Whole-cell protein extracts were subjected to 8% SDS-PAGE and immunoblot detection of LDLR and the FLAG epitope on PCSK9. Mature LDLR as well as a precursor form (pLDLR) were detected (arrows). TfR was detected as a control for loading. Secondary IRDye800-labeled antibodies were used and blots were imaged on a LI-COR Odyssey infrared system. B: SV-589 and HepG2 cells were cultured as in (A) in the continuous presence of E64 (150 μM) to inhibit endolysosomal cysteine protease activity. Cells were incubated for 1 h with Alexa488-labeled PCSK9-D374Y (1 μg/ml) and collected for FACS analysis as described in Materials and Methods. A lower threshold for positive staining was determined based on cells not incubated with fluorescent PCSK9. Results shown represent the mean and standard deviation from three separate experiments.

High cellular levels of sterols specifically suppress transcription of genes containing sterol-regulatory elements within their promoter regions, including *LDLR*, due to decreased proteolytic processing of the SREBP-2 transcription factor to its active form (1). Therefore, to assess whether PCSK9 cell-association and uptake was mediated by the LDLR in SV-589 fibroblasts we cultured SV-589 cells for more than 16 h in either sterol-depleting conditions (lipoprotein-deficient medium containing pravastatin) to induce LDLR mRNA and protein expression or medium supplemented with sterols (cholesterol and 25-hydroxycholesterol) to suppress LDLR expression. Cells were then incubated with FLAG-tagged wild-type PCSK9 (5 µg/ml) or PCSK9-D374Y (0.5 µg/ml) for 2 h to allow cell-association and uptake. Wild-type PCSK9 and PCSK9-D374Y were detected in blots of whole cell extracts derived from cells cultured under LDLR-inducing conditions whereas cell-associated PCSK9 was decreased to almost undetectable levels for cells cultured with sterols, mirroring the suppression of LDLR protein levels (**Fig. 2A**). To further quantify this effect, we also performed FACS analysis to assess sterol-dependent cell-association and uptake of Alexa488-labeled PCSK9-D374Y in SV-589 fibroblasts as well as HepG2 cells. Cell-associated fluorescence was decreased over 90% for both cell-lines cultured in the presence of sterols (Fig. 2B).

### Wild-type PCSK9 and mutant PCSK9-D374Y traffic to lysosomes in SV-589 fibroblasts

**Fig. 3.**
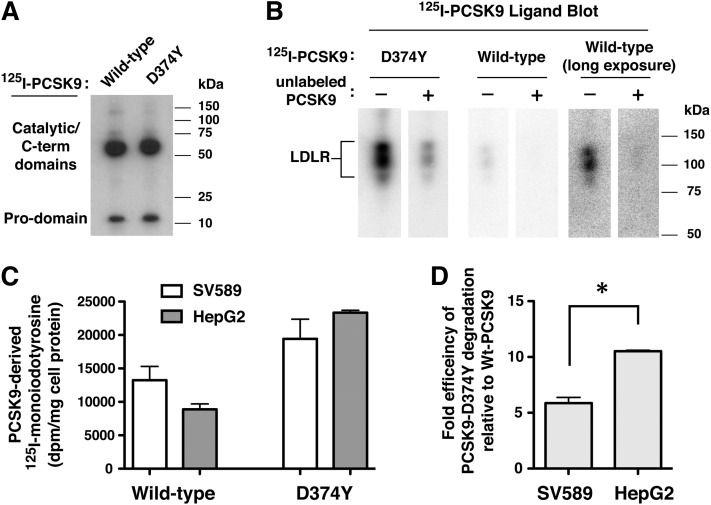
Lysosomal degradation of ^125^I-labeled wild-type PCSK9 and PCSK9-D374Y in SV-589 fibroblasts and HepG2 hepatoma cells. A: ^125^I-labeled wild-type PCSK9 and PCSK9-D374Y were subjected to 4–20% SDS-PAGE and imaged by autoradiography. B: Ligand blotting of ^125^I-labeled PCSK9 to LDLR. Membrane protein extracts from HEK293 cells transiently overexpressing LDLR were subjected to 8% SDS-PAGE under nonreducing conditions and blotted to nitrocellulose. Strips were incubated for 1 h with ^125^I-labeled PCSK9 proteins in the absence (−) or presence (+) of an excess of unlabeled PCSK9-D374Y as described in Materials and Methods. Following washes, dried blots were imaged on a Storm 860 Molecular Imager. C: SV-589 and HepG2 cells were cultured for more than 16 h in sterol-depleting Medium C prior to labeling for 1 h at 37°C with ^125^I-labeled wild-type PCSK9 (2 µg/ml) or 4-fold lower concentration of PCSK9-D374Y (0.5 µg/ml). Labeling medium was removed and replaced with label-free medium for 6 h at 37°C. Excreted TCA-soluble ^125^I-labeled PCSK9 degradation products (monoiodotyrosine) were isolated from the culture medium and measured by γ counting as described in Materials and Methods. D: The data in © are expressed as efficiency of PCSK9-D374Y degradation compared with that of wild-type PCSK9 for each cell-line. *P < 0.05 between the two groups (Student *t*-test).

To examine the fate of PCSK9 in SV-589 fibroblasts, we measured lysosomal degradation of internalized ^125^I-labeled wild-type PCSK9 and PCSK9-D374Y. For comparison purposes, we performed the same experiments in HepG2 cells, which have previously been shown to degrade ^125^I-labeled PCSK9 within lysosomes (28). Radioiodinated PCSK9 consisted of intact prodomain and catalytic/C-terminal domain segments (**Fig. 3A**) and showed a high specificity for binding to LDLRs, as demonstrated by ligand blotting of total membrane protein extracts from HEK293 cells transiently overexpressing LDLR (Fig. 3B). As expected, ^125^I-labeled PCSK9-D374Y displayed increased binding affinity for LDLRs compared with wild-type PCSK9 (Fig. 3B). For cellular degradation studies, SV-589 and HepG2 cells were pulse-labeled for 1 h with ^125^I-labeled wild-type PCSK9 (2 µg/ml) or PCSK9-D374Y (0.5 µg/ml) followed by a 6 h chase period. Excreted ^125^I-monoiodotyrosine (TCA-soluble), a catabolic product of lysosomal degradation of ^125^I-labeled proteins (38), was then measured in the culture medium as a determinant of lysosomal trafficking and degradation of internalized radioiodinated PCSK9. These values were corrected for nonlysosomal degradation, measured as TCA-soluble radioactivity generated in cells preincubated with 50 µM chloroquine (typically less than 10% of total counts). Internalized ^125^I-labeled wild-type PCSK9 was degraded in lysosomes of SV-589 fibroblasts at high levels comparable to that in HepG2 cells (Fig. 3C). Despite a 4-fold lower concentration used for cell labeling, ^125^I-PCSK9-D374Y was degraded at 2.6-fold and 1.5-fold higher levels than wild-type PCSK9 in HepG2 cells and SV-589 fibroblasts, respectively. Thus, the efficiency of PCSK9-D374Y degradation was approximately 10-fold higher than wild-type PCSK9 in HepG2 cells compared with approximately 6-fold in SV-589 cells (Fig. 3D). HepG2 cells and SV-589 fibroblasts internalized and degraded equivalent amounts of ^125^I-labeled C7 monoclonal antibody, which releases from the LDLR within the acidic environment of early endosomes (41), indicating that cell surface LDLR expression and receptor endocytosis were comparable between the two cell-lines (data not shown).

**Fig. 4.**
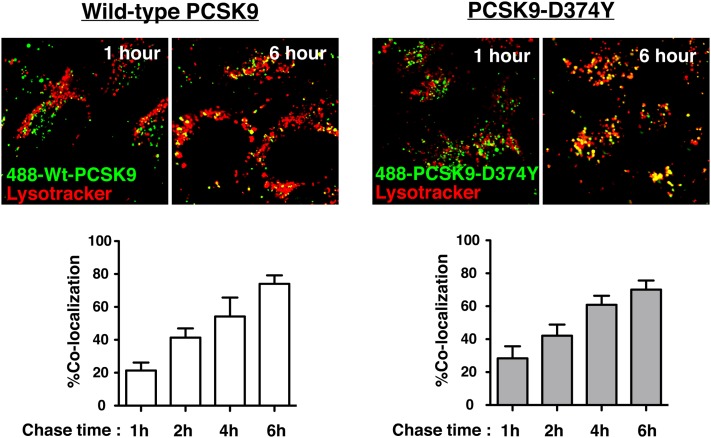
Wild-type PCSK9 and PCSK9-D374Y traffic to lysosomes in SV-589 fibroblasts. SV-589 cells seeded on 8-well chambered coverglass were cultured for more than 16 h in sterol-depleting Medium C in the presence of E64 (150 μM). Cells were labeled with Alexa488-labeled wild-type PCSK9 (30 μg/ml) or PCSK9-D347Y (5 µg/ml) for 1 h and chased up to 6 h in label-free Medium C containing E64. LysoTracker Red DND-99 was incubated at concentration 200 nM for 2 h prior the end of the chase period. Images were taken on Olympus FV1000 scanning confocal microscope. The percentage of colocalization of PCSK9 fluorescence with the lysosomal marker LysoTracker was quantified using Image J software from five or more fields encompassing more than 100 cells for each condition. Results shown are the mean and standard deviation from three separate experiments.

To further confirm lysosomal trafficking of PCSK9 proteins in SV-589 fibroblasts, we performed live-cell confocal microscopy analysis to assess colocalization of internalized Alexa488-labeled wild-type PCSK9 and PCSK9-D374Y with LysoTracker, a cell-stain specific for acidic late endosomes/lysosomes. Cells were first pulse-labeled with fluorescently-labeled PCSK9 proteins for 1 h in medium containing E64, an inhibitor of lysosomal cysteine proteases, then washed to remove unbound label and incubated for a further 6 h in the presence of E64. Internalized Alexa488-labeled PCSK9 was initially localized in diffuse punctate structures throughout the cytoplasm that over a 6 h time-period showed increased colocalization with LysoTracker (**Fig. 4**), indicating the trafficking of both wild-type PCSK9 and PCSK9-D374Y to lysosomes.

### Internalized mutant PCSK9-D374Y, but not wild-type PCSK9, enters a recycling pathway in SV-589 fibroblasts

**Fig. 5.**
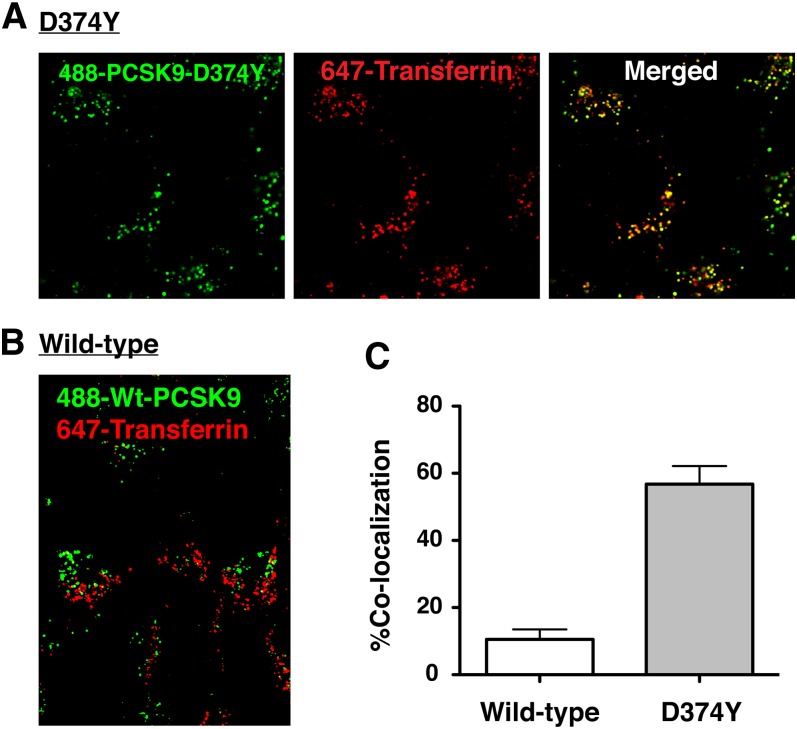
Trafficking of PCSK9-D374Y to endocytic recycling compartments in SV-589 cells. SV-589 cells seeded on 8-well chambered coverglass were cultured for more than 16 h in sterol-depleting Medium C in the presence of E64 (150 μM). Cells were labeled with 5 µg/ml AlexaFluor 488-labeled PCSK9-D374Y (A) or 30 µg/ml wild-type PCSK9 (B) along with 100 µg/ml Alexa647-labeled transferrin for 1 h in Medium A. Labeling medium was replaced with label-free Medium A containing E64 for a 2 h chase period and images were taken on Olympus FV1000 scanning confocal microscope. C: The percentage of colocalization of PCSK9 fluorescence with that of the endocytic recycling compartment marker transferrin was quantified using Image J software from five or more fields encompassing more than 100 cells for each condition. Results shown are the mean and standard deviation from three separate experiments.

Relative to the degradation of wild-type PCSK9, the degradation of ^125^I-labeled PCSK9-D374Y appeared to be less efficient in SV-589 fibroblasts compared with HepG2 cells (Fig. 3D), suggesting that a lower proportion of internalized PCSK9-D374Y traffics to lysosomes in fibroblasts. To assess potential cell surface recycling of this LDLR ligand in SV-589 fibroblasts, we performed live-cell confocal microscopy analysis to determine colocalization of internalized Alexa488-labeled PCSK9-D374Y with Alexa647-labeled transferrin (Tf), a marker of the endocytic recycling compartment (ERC). Cells were pulsed with fluorophore-labeled PCSK9-D374Y and Tf for 1 h, followed by a 4 h chase period to allow movement out of early endosomal compartments. Following this treatment, PCSK9-D374Y was robustly colocalized with Tf in punctate cytoplasmic structures, indicating that a substantial proportion of internalized PCSK9-D374Y trafficked to the ERC in SV-589 fibroblasts (**Fig. 5A, C**). In contrast, there was minimal ERC localization of internalized Alexa488-labeled wild-type PCSK9 under the same conditions (Fig. 5B, C), consistent with predominant trafficking of wild-type PCSK9 to lysosomes.

**Fig. 6.**
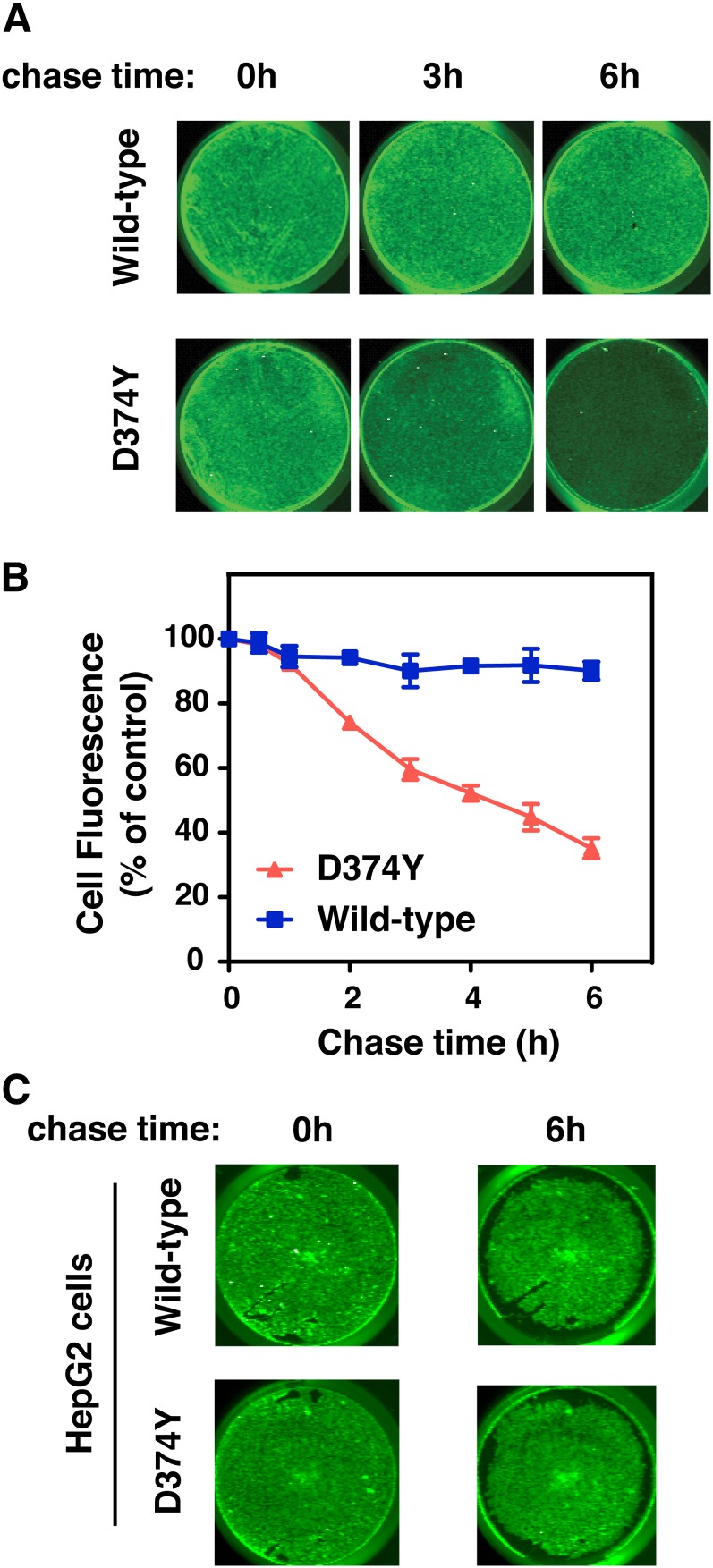
A large proportion of internalized PCSK9-D374Y recycles to the cell surface in SV-589 fibroblasts. A: SV-589 cells seeded on 24-well plates were cultured for more than 16 h in sterol-depleting Medium C in the presence of E64 (150 μM). Wild-type PCSK9 or PCSK9-D347Y covalently modified with thiol-cleavable (S-S)-biotin was preincubated with IRDye800CW streptavidin in Medium A for 1 h and added to cells for 1 h at 37°C. Labeling medium was replaced with label-free Medium A containing the noncell-permeable reducing agent TCEP (20 mM) and E64 (150 μM) and incubated at 37°C for a 6 h chase period. Plates were scanned directly on a LI-COR Odyssey infrared system at the indicated time points to measure remaining cell-associated fluorescence. Individual wells from a representative experiment are shown. B: Quantification of PCSK9-S-S-biotin-800Streptavidin recycling based on total fluorescence following continuous exposure to TCEP. Values were normalized to cell density measured using a DNA stain (DRAQ5). Results shown are expressed relative to the start of the 6 h chase period and represent the mean and standard deviation of three separate experiments. C: Lack of cell surface recycling of internalized PCSK9-D374Y in HepG2 cells. HepG2 cells were cultured and treated as in (A). Plates were scanned directly on a LI-COR Odyssey infrared system at the indicated time points to measure remaining cell-associated PCSK9 fluorescence. Individual wells are shown from a representative experiment repeated two other times with similar results.

To quantify the amount of cell surface recycling of wild-type PCSK9 and PCSK9-D374Y in SV-589 fibroblasts, we turned to a recycling assay using PCSK9 covalently modified with thiol-cleavable biotin (S-S-biotin). Biotinylated PCSK9 was labeled in solution with IRDye800-labeled streptavidin and added to cells for 1 h to allow uptake of fluorescent PCSK9-S-S-biotin–800streptavidin complexes. To remove surface fluorescence, cells were washed in a buffer containing the reducing agent TCEP, which is highly charged and does not cross cell membranes (42). Labeled cells were then cultured in medium containing TCEP to cleave the releasable fluorescent tag from PCSK9 complexes returning to the cell surface, and time-dependent loss of cell-associated fluorescence was quantified as a measure of endocytic recycling (**Fig. 6A**). Culture medium also contained E64 to prevent potential loss of cell-associated fluorescence due to lysosomal degradation. Following a 6 h period, greater than 50% of internalized PCSK9-D374Y complexes recycled to the cell surface versus less than 10% for wild-type PCSK9 (Fig. 6B), further demonstrating the differential trafficking of wild-type PCSK9 and PCSK9-D374Y in SV-589 fibroblasts. Under identical assay conditions, there was less than 10% loss of fluorescence for internalized wild-type PCSK9 or PCSK9-D374Y in HepG2 cells (Fig. 6C), consistent with predominant trafficking of PCSK9 to lysosomes in hepatic cells.

## DISCUSSION

The degree of LDLR degradation in response to exogenous PCSK9 varies among cell-types in culture. PCSK9 efficiently targets the LDLR for lysosomal degradation in liver-derived cells but not immortalized fibroblast cell-lines, despite LDLR-dependent uptake into endolysosomal compartments (5, 35). It is unknown whether internalized PCSK9 remains associated with LDLR in resistant cell-types, or if it dissociates within early endosomes prior to LDLR recycling. In the current study, we report that PCSK9 is released from the LDLR and delivered to lysosomes as a dissociated ligand in SV-589 cells, a line of SV40-transformed human skin fibroblasts with a robust PCSK9-resistant phenotype. A gain-of-function D374Y mutant PCSK9 with increased binding affinity to LDLR was capable of directing LDLR degradation in a limited manner in these cells; however, much of the internalized PCSK9-D374Y was recycled to the cell surface, presumably still bound to the LDLR. We conclude that two factors diminish PCSK9 activity in SV-589 fibroblasts: *i*) an increased dissociation from the LDLR in early endosomes, and *ii*) a decreased ability of bound PCSK9 to inhibit LDLR recycling.

Tissue specificity to circulating PCSK9 has been observed in mice, with PCSK9 activity toward LDLRs being suppressed in extrahepatic tissues such as adrenal gland and kidney (27, 43, 44). Compared with liver, there was decreased accumulation of injected ^125^I-labeled PCSK9 in adrenal gland in a recent study (45), suggesting that PCSK9 uptake was inhibited in this tissue, perhaps due to a blocking effect on LDLR binding exerted by extracellular annexin A2 (44, 46). In contrast, cell-association and uptake of exogenous wild-type PCSK9 and gain-of-function PCSK9-D374Y was normal in PCSK9-resistant SV-589 fibroblasts (Fig. 1), thus implicating an intracellular mechanism of PCSK9 inhibition. In SV-589 cells treated with sterols to suppress LDLR mRNA and protein expression, the uptake of PCSK9 was reduced to almost undetectable levels (Fig. 2). This is in agreement with a previous report in which an unambiguous role of the LDLR for PCSK9 internalization was determined through the use of MEF cells derived from wild-type or *Ldlr*^−/−^ mice (5). Missense mutations in *PCSK9* (D374Y) or *LDLR* (H306Y) that improve specific bond interactions at the PCSK9-LDLR interface increased cellular uptake of PCSK9 accordingly (5, 19). Injection studies in mice demonstrated that the plasma clearance rate of gain-of-function PCSK9-D374Y was increased compared with that of wild-type PCSK9, supporting a direct role of LDLR in PCSK9 uptake in vivo (27). A recent report suggests that PCSK9 degradation in hepatic cells is directly mediated by an interaction with amyloid precursor-like protein 2 (APLP2), a ubiquitously expressed cell surface protein known to be involved in lysosomal trafficking of MHC class I K^d^ molecules (47). In this model, constitutive lysosomal trafficking of PCSK9 is mediated by APLP2, and LDLR is degraded as a consequence of binding to PCSK9; LDLR-dependence of PCSK9 uptake is explained by an unappreciated role of the LDLR in the organization of endocytic adaptor proteins and receptor clustering at the plasma membrane. However, it should be noted that LDLR is dispersed on the plasma membrane in hepatocytes but clustered in coated pits in fibroblasts (48). The viability of *Ldlr^−/−^* mice also argues against a general role of the LDLR in adaptor protein recruitment and receptor clustering in coated pits. PCSK9 association with APLP2 requires the C-terminal domain of PCSK9 (47) and could explain antigen-mediated clearance of therapeutic blocking antibodies targeting the PCSK9 catalytic domain (49). However, this pathway may not represent a predominant means of PCSK9 internalization in cells and tissues when LDLR binding is not inhibited.

In the current study, we found that lysosomal degradation of ^125^I-labeled PCSK9-D374Y was 2.6-fold higher than wild-type PCSK9 in HepG2 cells despite being added to culture medium at a 4-fold lower concentration (Fig. 3C). Thus, the efficiency of PCSK9-D374Y degradation was approximately 10-fold higher than wild-type PCSK9, in accordance with a 10- to 25-fold increased binding affinity to the LDLR at physiological pH. PCSK9-D374Y treatment (2 µg/ml) reduced cell surface LDLRs in SV-589 cells by approximately 50% (Fig. 1B), indicating that this gain-of-function mutant PCSK9 was able to direct LDLR degradation, albeit at lower levels than in HepG2 cells. Decreased LDLR degradation activity by PCSK9-D374Y in SV-589 fibroblasts could be due to inefficient lysosomal targeting of LDLRs by bound PCSK9. Indeed, internalized PCSK9-D374Y was shown to traffic to either lysosomes (Fig. 4) or to transferrin-positive endocytic recycling compartments in SV-589 fibroblasts (Fig. 5). We did not observe localization of fluorophore-labeled PCSK9-D374Y in endocytic recycling compartments of HepG2 cells (data not shown).

One potential mechanism for LDLR degradation mediated by PCSK9 is through interference with a closed conformation of the LDLR in acidic endocytic compartments (28–30). *LDLR* mutations that negatively affect the closed conformation lead to defective LDLR recycling and increased degradation in cultured cells (50–52). Surprisingly, a large proportion (>50%) of internalized PCSK9-D374Y recycled to the cell surface in SV-589 fibroblasts, presumably still bound to the LDLR (Fig. 6B). This suggests that bound PCSK9 does not greatly affect the open-to-closed conformational switch in SV-589 cells or that other factors are required for efficient degradation of LDLR. In fibroblasts, endocytosis of LDLR in clathrin-coated vesicles is predominantly mediated by the adaptor protein Disabled-2 (53), whereas ARH is required for endocytosis of LDLR in hepatic cells (54). ARH was shown to be essential for PCSK9-mediated LDLR degradation in primary mouse hepatocytes (5) but not in primary human fibroblasts (35). There is evidence that membrane receptors and their ligands can be sorted into clathrin-coated vesicles that merge with distinct subpopulations of early endosomes depending on the adaptor proteins involved (55, 56). The divergence of pathways for PCSK9-D374Y bound to LDLR in SV-589 fibroblasts (lysosomal versus recycling) may reflect less stringent adaptor-mediated sorting in fibroblasts compared with hepatocytes.

Importantly, we found that internalized ^125^I-labeled wild-type PCSK9 was degraded in lysosomes at comparable levels in HepG2 cells and SV-589 fibroblasts (Fig. 3C). Since cell surface LDLR levels were minimally decreased in SV-589 cells in response to PCSK9 (Fig. 1), this indicates that the majority of internalized PCSK9 was released from the LDLR, which was then free to recycle to the cell surface. In further support of this conclusion, we demonstrated that wild-type PCSK9 trafficked to lysosomes in SV-589 fibroblasts (Fig. 4) and did not recycle to the cell surface (Fig. 6A, B). The finding that PCSK9 releases from LDLRs in acidic early endosomes of certain cell-types was unanticipated since in vitro studies have shown that the binding affinity of PCSK9 to LDLR is greatly enhanced (>100-fold) at acidic pH (<5.5) as compared with physiological pH 7.4 (12, 13). However, a study of ^125^I-labeled PCSK9 binding to LDLR showed that the most pronounced increase occurred over a pH range (6.0–5.2) typical of late endosomal compartments (9). Therefore the pH effect could be more modest in early endosomes that are mildly acidic (5.9–6.8) and perhaps outweighed by other factors that promote PCSK9 dissociation from LDLR.

The LDLR EGF-A domain contains a noncanonical Ca^2+^ binding site (57) and Ca^2+^ coordination is required for PCSK9 association (9). Interestingly, the K_D_ for the Ca^2+^ binding in the EGF-A domain was determined to be approximately 50 µM, leading to the suggestion that the EGF-A Ca^2+^ site may be titrated in the low Ca^2+^ environment of early endosomes (∼10–50 µM) (57). This could in turn trigger PCSK9 dissociation dependent on other factors such as pH and *PCSK9* mutations that affect LDLR binding affinity. It is possible the pH and Ca^2+^ environment in early endosomes of hepatocytes strongly favors persistent PCSK9 binding to LDLR, whereas alterations in these parameters (higher pH and/or lower Ca^2+^) favor PCSK9 dissociation in resistant cells. The luminal ionic composition in the endolysosomal compartment can vary in an incremental manner between cell-types, largely dependent on differential expression of membrane ion pumps and channels (58, 59). This could dictate a range of potencies for PCSK9-mediated LDLR degradation across various cell-types and tissues, with hepatocytes and immortalized fibroblasts representing different ends of the spectrum. Dissociation of wild-type PCSK9 from the LDLR in early endosomes of SV-589 fibroblasts was likely not absolute, as high concentrations (20 µg/ml) significantly lowered cell surface LDLRs by approximately 25% (Fig. 1B).

For PCSK9 to direct LDLR trafficking it must remain bound to the receptor in early endosomes, a major site for the sorting of membrane components destined for recycling to the cell surface from those targeted for degradation in lysosomes. Our findings identify dissociation from the LDLR in early endosomal compartments as a determining mechanism for cell-type-specific resistance to PCSK9, a protein of major clinical importance for its ability to mediate LDLR degradation in liver. Continued study of the mechanisms regulating PCSK9 release from recycling LDLRs in PCSK9-resistant fibroblasts could shed light on factors that promote persistent binding in hepatocytes, eventually leading to LDLR degradation.
